# 16α,17α-Ep­oxy-17β-(1*H*-imidazol-1-yl)androst-4-en-3-one monohydrate

**DOI:** 10.1107/S1600536812029479

**Published:** 2012-07-07

**Authors:** A. G. Anitha, R. Hema, Ranju Bansal, Sridhar Thota, S. Rizwana Begum

**Affiliations:** a Department of Physics, Seethalakshmi Ramaswami College(Autonomous), Tiruchirappalli 620 002, India; b University Institute of Pharmaceutical Sciences, Panjab University, Chandigarh 160 014, India

## Abstract

In the title compound, C_22_H_28_N_2_O_2_·H_2_O, rings *B* and *C* adopt chair conformations. Ring *A* adopts an envelope conformation, with the non-fused C atom adjacent to the fused C atom bearing a methyl group as the flap atom. Ring *D* also adopts an envelope conformation, with the fused C atom not bearing a methyl group as the flap atom. The water mol­ecule links the mol­ecules *via* O—H⋯O and O—H⋯N hydrogen bonds, forming zigzag chains which run parallel to the *c* axis. Weak C—H⋯O inter­actions also occur.

## Related literature

For background information on steroid activity, see: Duax & Norton (1975 ▶). For conformational analysis, see: Altona *et al.* (1968 ▶); Cremer & Pople (1975 ▶). For details of the determination of the absolute configuration, see: Bansal *et al.* (2012 ▶).
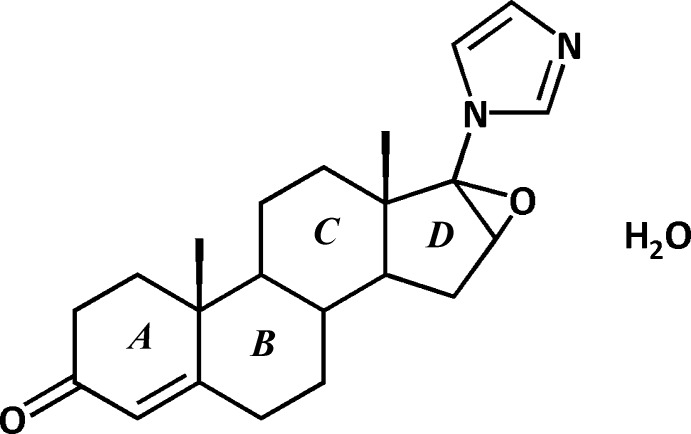


## Experimental

### 

#### Crystal data

C_22_H_28_N_2_O_2_·H_2_O
*M* *_r_* = 370.48
Orthorhombic, 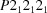

*a* = 9.7813 (2) Å
*b* = 13.5885 (3) Å
*c* = 14.2698 (3) Å
*V* = 1896.64 (7) Å^3^
*Z* = 4
Mo *K*α radiation
μ = 0.09 mm^−1^
*T* = 293 K
0.30 × 0.20 × 0.20 mm

#### Data collection

Bruker Kappa APEXII CCD diffractometer
Absorption correction: multi-scan (*SADABS*; Bruker, 2004) ▶ *T*_min_ = 0.975, *T*_max_ = 0.983
10371 measured reflections
3331 independent reflections
2807 reflections with *I* > 2σ(*I*)
*R*_int_ = 0.029

#### Refinement

*R*[*F* ^2^ > 2σ(*F* ^2^)] = 0.033
*wR*(*F* ^2^) = 0.083
*S* = 1.06
3331 reflections
246 parameters
H-atom parameters constrained
Δρ_max_ = 0.12 e Å^−3^
Δρ_min_ = −0.16 e Å^−3^

### 

Data collection: *APEX2* (Bruker, 2004 ▶); cell refinement: *APEX2* and *SAINT* (Bruker, 2004 ▶); data reduction: *SAINT* and *XPREP* (Bruker, 2004 ▶); program(s) used to solve structure: *SIR92* (Altomare *et al.*, 1994 ▶); program(s) used to refine structure: *SHELXL97* (Sheldrick, 2008 ▶); molecular graphics: *PLATON* (Spek, 2009 ▶); software used to prepare material for publication: *SHELXL97*.

## Supplementary Material

Crystal structure: contains datablock(s) I, global. DOI: 10.1107/S1600536812029479/go2059sup1.cif

Structure factors: contains datablock(s) I. DOI: 10.1107/S1600536812029479/go2059Isup2.hkl

Additional supplementary materials:  crystallographic information; 3D view; checkCIF report

## Figures and Tables

**Table 1:** Hydrogen-bond geometry (Å, °)

*D*—H⋯*A*	*D*—H	H⋯*A*	*D*⋯*A*	*D*—H⋯*A*
O3—H31⋯N2	0.90	1.99	2.890 (3)	174
O3—H32⋯O1^i^	0.90	2.33	3.202 (3)	163
C20—H20⋯O3^ii^	0.93	2.43	3.208 (4)	142

Symmetry codes: (i) 
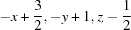
; (ii) 
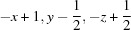
.

**Fig. 1.**
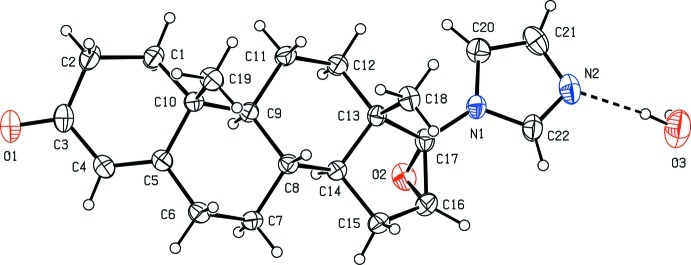
View of the molecule of the title compound showing the atom-labelling scheme. Displacement ellipsoids are drawn at the 30% probability level. H atoms are represented by circles of arbitrary radii.

**Fig. 2.**
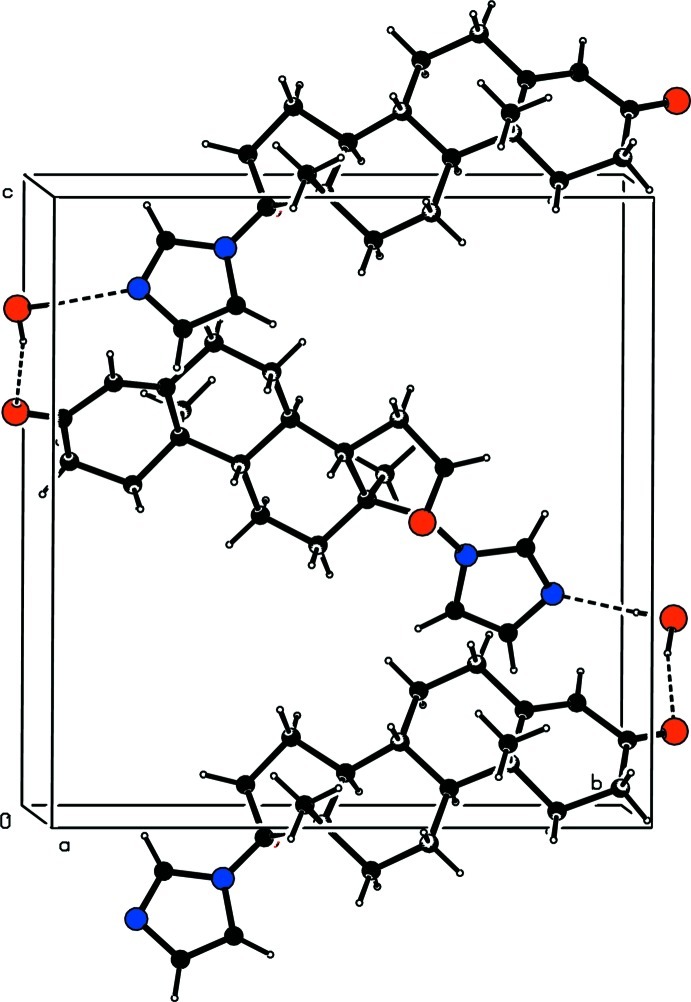
View of the zig-zag hydrogen bonded chain running parallel to the c-axis.

## supplementary crystallographic information

### Comment

It is well known that minor changes in the basic composition of steroids significantly alter their biological activities (Duax and Norton, 1975).

The structure determination of C_22_H_28_N_2_O_2_.H_2_O , (I), was undertaken to investigate the conformation of the fused ring system. The puckering parameters in (I), ring-B: Q = 0.534 (2) Å, θ = 3.9 (2)°, ring-C: Q = 0.589 (2) Å, θ = 6.19 (19)°; (Cremer and Pople, 1975) show that rings B and C adopt chair confomation. The C4—C5-(Csp2-Csp2) distance of 1.336 (3) Å confirms the localization of a double bond at this position. Due to this double bond the environment of atom C5 is planar, and hence ring A is slightly distorted towards an envelope conformation with puckering parameters, Q = 0.435 (3) Å, θ = 56.4 (4)°, φ = 18.7 (4)°, with C1 being the flap. The five-membered ring-D exhibits an envelope conformation, with C14 being the flap, with pseudorotation parameter (P =14.7 (3)° and τ =38.7 (1)°), (Altona *et al.*, 1968). The dihedral angle between the plane of imidazole moiety and the mean plane of rings A, B, C and D is 11.83 (9)°. The substitution of O2 between C17 and C16 does not affect the normal value of exocyclic angle of C16—C17—N1(121.8 (2)°). The water molecule links the molecules, via O3—H31···N2(within the asymmetric unit) and O3—H32···O1(3/2-x,2-y,-1/2+z hydrogen bonds, to form a zig-zag chains which run parallel to the c-axis. molecules. The molecular packing is also stabilized by weak C20—H20···O3( 1-x,-1/2+y,-1/2-z)) intermolecular interactions. Details of the determination of the absolute configuration can be found in (Bansal, *et al.* 2012).

### Experimental

A mixture of imidazole(1 g, 2.75 mmol) and anhydrous potassium carbonate(1 g) was stirred and refluxed in ethyl methyl ketone(50) ml for one hour. 16α/β-bromo-4-androstene-3, 17-dione(0.4 g, 1.09 mmol) was added to the reaction mixture and further refluxed for 3 h with continous stirring. The completion of reaction was monitored by TLC. The slurry was cooled, filtered and excess of solvent was removed under reduced pressure to obtain an oily residue. Iced water was added to the oily residue and it was allowed to stand overnight. The solid obtained was filtered, washed with water, dried and crystallized from acetone and hexane to afford the title compound(0.25 g, 64.78%), mp 419–420K.

### Refinement

All H atoms attached to C atoms were refined as riding atoms. The methyl H atoms were constrained to an ideal geometry (C—H = 0.98 Å) with *U*_iso_(H) = 1.5*U*_eq_(C), but were allowed to rotate freely about the C—C bonds. All remaining H atoms were placed in geometrically idealized positions (C—H = 0.95–1.00 Å) with *U*_iso_(H) = 1.2*U*_eq_(C). The water H atoms, which were initially located on a difference Fourier map. The O–H distance was then restrained to a distancet of 0.900 (2)Å and then, in the final stages of the refinement, refined as riding atoms with *U*_iso_(H) = 1.5*U*_eq_(O). These positions were checked in a final difference Fourier and found to be satisfactory.

Friedel Pairs were merged.

### Crystal data

**Table:**

C_22_H_28_N_2_O_2_·H_2_O	*F*(000) = 800
*M**_r_* = 370.48	*D*_x_ = 1.297 Mg m^−^^3^
Orthorhombic, *P*2_1_2_1_2_1_	Mo *K*α radiation, λ = 0.71073 Å
Hall symbol: P 2ac 2ab	Cell parameters from 3481 reflections
*a* = 9.7813 (2) Å	θ = 4.0–29.1°
*b* = 13.5885 (3) Å	µ = 0.09 mm^−^^1^
*c* = 14.2698 (3) Å	*T* = 293 K
*V* = 1896.64 (7) Å^3^	Block, colourless
*Z* = 4	0.30 × 0.20 × 0.20 mm

### Data collection

**Table:**

Bruker Kappa APEXII CCD diffractometer	3331 independent reflections
Radiation source: fine-focus sealed tube	2807 reflections with *I* > 2σ(*I*)
Graphite monochromator	*R*_int_ = 0.029
ω and φ scan	θ_max_ = 25.0°, θ_min_ = 2.5°
Absorption correction: multi-scan (*SADABS*; Bruker, 2004)	*h* = −11→11
*T*_min_ = 0.975, *T*_max_ = 0.983	*k* = −13→16
10371 measured reflections	*l* = −16→16

### Refinement

**Table:**

Refinement on *F*^2^	Primary atom site location: structure-invariant direct methods
Least-squares matrix: full	Secondary atom site location: difference Fourier map
*R*[*F*^2^ > 2σ(*F*^2^)] = 0.033	Hydrogen site location: inferred from neighbouring sites
*wR*(*F*^2^) = 0.083	H-atom parameters constrained
*S* = 1.06	*w* = 1/[σ^2^(*F*_o_^2^) + (0.0416*P*)^2^ + 0.2407*P*] where *P* = (*F*_o_^2^ + 2*F*_c_^2^)/3
3331 reflections	(Δ/σ)_max_ < 0.001
246 parameters	Δρ_max_ = 0.12 e Å^−^^3^
0 restraints	Δρ_min_ = −0.16 e Å^−^^3^

### Special details

**Table:**

Geometry. All esds (except the esd in the dihedral angle between two l.s. planes) are estimated using the full covariance matrix. The cell esds are taken into account individually in the estimation of esds in distances, angles and torsion angles; correlations between esds in cell parameters are only used when they are defined by crystal symmetry. An approximate (isotropic) treatment of cell esds is used for estimating esds involving l.s. planes.
Refinement. Refinement of *F*^2^ against ALL reflections. The weighted *R*-factor *wR* and goodness of fit *S* are based on *F*^2^, conventional *R*-factors *R* are based on *F*, with *F* set to zero for negative *F*^2^. The threshold expression of *F*^2^ > σ(*F*^2^) is used only for calculating *R*-factors(gt) *etc*. and is not relevant to the choice of reflections for refinement. *R*-factors based on *F*^2^ are statistically about twice as large as those based on *F*, and *R*- factors based on ALL data will be even larger.

### Fractional atomic coordinates and isotropic or equivalent isotropic displacement parameters (Å^2^)

**Table:**

	*x*	*y*	*z*	*U*_iso_*/*U*_eq_	
O1	0.6588 (2)	−0.04599 (13)	0.64733 (14)	0.0679 (6)	
O2	0.76907 (16)	0.62764 (13)	0.47612 (12)	0.0530 (5)	
N1	0.5710 (2)	0.71088 (13)	0.41686 (14)	0.0414 (5)	
N2	0.5333 (2)	0.85703 (17)	0.35422 (18)	0.0605 (6)	
C1	0.5023 (3)	0.15748 (17)	0.52622 (17)	0.0468 (6)	
H11	0.4263	0.1669	0.4835	0.056*	
H12	0.5860	0.1664	0.4907	0.056*	
C2	0.4982 (3)	0.05208 (19)	0.56319 (19)	0.0543 (7)	
H21	0.4093	0.0393	0.5907	0.065*	
H22	0.5110	0.0066	0.5115	0.065*	
C3	0.6069 (3)	0.03508 (18)	0.63520 (18)	0.0494 (6)	
C4	0.6411 (3)	0.11847 (18)	0.69325 (16)	0.0447 (6)	
H4	0.7049	0.1090	0.7407	0.054*	
C5	0.5874 (2)	0.20829 (17)	0.68355 (15)	0.0367 (5)	
C6	0.6152 (3)	0.28656 (17)	0.75530 (15)	0.0450 (6)	
H61	0.6823	0.2624	0.7998	0.054*	
H62	0.5317	0.3006	0.7895	0.054*	
C7	0.6676 (3)	0.38044 (17)	0.71115 (15)	0.0423 (6)	
H71	0.7580	0.3690	0.6855	0.051*	
H72	0.6752	0.4309	0.7589	0.051*	
C8	0.5733 (2)	0.41648 (16)	0.63353 (15)	0.0348 (5)	
H8	0.4852	0.4358	0.6606	0.042*	
C9	0.5502 (2)	0.33505 (16)	0.56073 (14)	0.0332 (5)	
H9	0.6410	0.3197	0.5357	0.040*	
C10	0.4955 (2)	0.23647 (17)	0.60221 (14)	0.0363 (5)	
C11	0.4670 (3)	0.37260 (18)	0.47661 (15)	0.0454 (6)	
H111	0.4626	0.3210	0.4297	0.054*	
H112	0.3743	0.3860	0.4971	0.054*	
C12	0.5255 (3)	0.46578 (17)	0.43116 (15)	0.0426 (6)	
H121	0.6131	0.4510	0.4027	0.051*	
H122	0.4641	0.4884	0.3823	0.051*	
C13	0.5433 (2)	0.54667 (17)	0.50467 (14)	0.0337 (5)	
C14	0.6352 (2)	0.50357 (15)	0.58250 (15)	0.0346 (5)	
H14	0.7154	0.4772	0.5499	0.042*	
C15	0.6875 (3)	0.59235 (17)	0.63698 (17)	0.0461 (6)	
H151	0.6211	0.6144	0.6829	0.055*	
H152	0.7731	0.5778	0.6684	0.055*	
C16	0.7067 (3)	0.66715 (19)	0.56080 (18)	0.0493 (6)	
H16	0.7186	0.7368	0.5768	0.059*	
C17	0.6241 (2)	0.63942 (16)	0.48021 (16)	0.0384 (5)	
C18	0.4045 (3)	0.58323 (18)	0.53973 (17)	0.0448 (6)	
H181	0.3533	0.6095	0.4881	0.067*	
H182	0.4181	0.6337	0.5859	0.067*	
H183	0.3551	0.5294	0.5672	0.067*	
C19	0.3478 (2)	0.24693 (19)	0.63881 (17)	0.0480 (6)	
H191	0.3179	0.1852	0.6643	0.072*	
H192	0.2887	0.2656	0.5881	0.072*	
H193	0.3450	0.2965	0.6867	0.072*	
C20	0.5241 (3)	0.6948 (2)	0.32764 (17)	0.0504 (7)	
H20	0.5108	0.6345	0.2983	0.060*	
C21	0.5013 (3)	0.7846 (2)	0.29144 (19)	0.0590 (7)	
H211	0.4680	0.7961	0.2314	0.071*	
C22	0.5740 (3)	0.80970 (18)	0.4289 (2)	0.0517 (7)	
H221	0.6019	0.8403	0.4840	0.062*	
O3	0.6304 (3)	1.05448 (17)	0.31888 (16)	0.0989 (9)	
H31	0.5947	0.9940	0.3274	0.148*	
H32	0.6767	1.0425	0.2655	0.148*	

### Atomic displacement parameters (Å^2^)

**Table:**

	*U*^11^	*U*^22^	*U*^33^	*U*^12^	*U*^13^	*U*^23^
O1	0.0795 (14)	0.0405 (10)	0.0838 (13)	0.0127 (10)	0.0054 (12)	0.0068 (10)
O2	0.0378 (9)	0.0550 (11)	0.0663 (11)	−0.0018 (8)	0.0074 (8)	0.0142 (10)
N1	0.0427 (11)	0.0342 (11)	0.0474 (11)	−0.0017 (9)	0.0037 (10)	0.0052 (9)
N2	0.0583 (14)	0.0456 (13)	0.0776 (16)	0.0053 (12)	0.0054 (13)	0.0170 (13)
C1	0.0590 (16)	0.0353 (13)	0.0461 (13)	−0.0051 (12)	−0.0052 (13)	−0.0019 (11)
C2	0.0671 (18)	0.0346 (13)	0.0613 (16)	−0.0060 (13)	−0.0025 (15)	−0.0037 (12)
C3	0.0546 (16)	0.0367 (14)	0.0568 (15)	0.0027 (12)	0.0117 (14)	0.0077 (12)
C4	0.0430 (13)	0.0444 (14)	0.0467 (13)	0.0047 (12)	−0.0028 (12)	0.0068 (12)
C5	0.0323 (11)	0.0395 (13)	0.0384 (12)	−0.0044 (10)	0.0018 (10)	0.0075 (10)
C6	0.0550 (15)	0.0439 (14)	0.0360 (11)	−0.0014 (12)	−0.0084 (11)	0.0059 (11)
C7	0.0499 (15)	0.0389 (13)	0.0381 (12)	−0.0059 (12)	−0.0113 (11)	0.0005 (11)
C8	0.0375 (12)	0.0358 (12)	0.0310 (11)	−0.0012 (10)	0.0000 (10)	−0.0001 (9)
C9	0.0376 (12)	0.0312 (11)	0.0310 (11)	−0.0010 (10)	0.0008 (10)	−0.0001 (9)
C10	0.0390 (12)	0.0344 (12)	0.0353 (11)	−0.0025 (11)	−0.0037 (10)	0.0013 (9)
C11	0.0654 (15)	0.0370 (13)	0.0339 (11)	−0.0078 (12)	−0.0110 (12)	−0.0001 (10)
C12	0.0571 (15)	0.0384 (13)	0.0322 (11)	−0.0018 (12)	−0.0055 (11)	0.0025 (10)
C13	0.0361 (12)	0.0311 (11)	0.0339 (11)	−0.0009 (10)	0.0030 (10)	0.0020 (10)
C14	0.0344 (11)	0.0343 (12)	0.0352 (11)	−0.0010 (10)	−0.0001 (10)	−0.0018 (10)
C15	0.0514 (15)	0.0392 (13)	0.0476 (13)	−0.0080 (12)	−0.0094 (12)	0.0002 (12)
C16	0.0494 (14)	0.0386 (14)	0.0599 (15)	−0.0079 (12)	−0.0053 (14)	−0.0016 (12)
C17	0.0348 (12)	0.0339 (13)	0.0465 (13)	0.0028 (10)	0.0042 (11)	0.0020 (11)
C18	0.0400 (13)	0.0453 (14)	0.0492 (13)	0.0029 (11)	0.0070 (11)	0.0055 (11)
C19	0.0411 (13)	0.0515 (15)	0.0513 (14)	−0.0043 (12)	−0.0016 (12)	0.0103 (13)
C20	0.0555 (16)	0.0520 (16)	0.0438 (13)	−0.0005 (13)	0.0051 (12)	0.0039 (12)
C21	0.0570 (17)	0.0672 (19)	0.0527 (14)	0.0087 (15)	0.0071 (14)	0.0224 (15)
C22	0.0521 (15)	0.0351 (14)	0.0680 (17)	0.0009 (12)	0.0010 (15)	0.0048 (13)
O3	0.142 (2)	0.0577 (13)	0.0970 (16)	0.0040 (16)	0.0123 (17)	0.0052 (13)

### Geometric parameters (Å, º)

**Table:**

O1—C3	1.226 (3)	C9—H9	0.9800
O2—C17	1.428 (3)	C10—C19	1.542 (3)
O2—C16	1.456 (3)	C11—C12	1.533 (3)
N1—C22	1.354 (3)	C11—H111	0.9700
N1—C20	1.371 (3)	C11—H112	0.9700
N1—C17	1.425 (3)	C12—C13	1.529 (3)
N2—C22	1.307 (3)	C12—H121	0.9700
N2—C21	1.367 (4)	C12—H122	0.9700
C1—C2	1.527 (3)	C13—C17	1.528 (3)
C1—C10	1.527 (3)	C13—C18	1.530 (3)
C1—H11	0.9700	C13—C14	1.544 (3)
C1—H12	0.9700	C14—C15	1.524 (3)
C2—C3	1.496 (4)	C14—H14	0.9800
C2—H21	0.9700	C15—C16	1.500 (3)
C2—H22	0.9700	C15—H151	0.9700
C3—C4	1.443 (4)	C15—H152	0.9700
C4—C5	1.336 (3)	C16—C17	1.455 (3)
C4—H4	0.9300	C16—H16	0.9800
C5—C6	1.501 (3)	C18—H181	0.9600
C5—C10	1.517 (3)	C18—H182	0.9600
C6—C7	1.512 (3)	C18—H183	0.9600
C6—H61	0.9700	C19—H191	0.9600
C6—H62	0.9700	C19—H192	0.9600
C7—C8	1.522 (3)	C19—H193	0.9600
C7—H71	0.9700	C20—C21	1.344 (4)
C7—H72	0.9700	C20—H20	0.9300
C8—C14	1.516 (3)	C21—H211	0.9300
C8—C9	1.534 (3)	C22—H221	0.9300
C8—H8	0.9800	O3—H31	0.90
C9—C11	1.538 (3)	O3—H32	0.90
C9—C10	1.559 (3)		
			
C17—O2—C16	60.59 (15)	C9—C11—H112	108.8
C22—N1—C20	106.5 (2)	H111—C11—H112	107.6
C22—N1—C17	125.9 (2)	C13—C12—C11	110.25 (17)
C20—N1—C17	127.1 (2)	C13—C12—H121	109.6
C22—N2—C21	104.5 (2)	C11—C12—H121	109.6
C2—C1—C10	114.4 (2)	C13—C12—H122	109.6
C2—C1—H11	108.7	C11—C12—H122	109.6
C10—C1—H11	108.7	H121—C12—H122	108.1
C2—C1—H12	108.7	C17—C13—C12	119.69 (18)
C10—C1—H12	108.7	C17—C13—C18	105.42 (18)
H11—C1—H12	107.6	C12—C13—C18	110.9 (2)
C3—C2—C1	111.3 (2)	C17—C13—C14	100.14 (17)
C3—C2—H21	109.4	C12—C13—C14	106.69 (18)
C1—C2—H21	109.4	C18—C13—C14	113.86 (18)
C3—C2—H22	109.4	C8—C14—C15	120.47 (18)
C1—C2—H22	109.4	C8—C14—C13	114.14 (18)
H21—C2—H22	108.0	C15—C14—C13	105.19 (17)
O1—C3—C4	121.9 (2)	C8—C14—H14	105.2
O1—C3—C2	122.0 (2)	C15—C14—H14	105.2
C4—C3—C2	116.0 (2)	C13—C14—H14	105.2
C5—C4—C3	124.5 (2)	C16—C15—C14	102.05 (18)
C5—C4—H4	117.7	C16—C15—H151	111.4
C3—C4—H4	117.7	C14—C15—H151	111.4
C4—C5—C6	120.3 (2)	C16—C15—H152	111.4
C4—C5—C10	122.9 (2)	C14—C15—H152	111.4
C6—C5—C10	116.77 (19)	H151—C15—H152	109.2
C5—C6—C7	112.03 (18)	C17—C16—O2	58.74 (14)
C5—C6—H61	109.2	C17—C16—C15	109.1 (2)
C7—C6—H61	109.2	O2—C16—C15	113.8 (2)
C5—C6—H62	109.2	C17—C16—H16	120.0
C7—C6—H62	109.2	O2—C16—H16	120.0
H61—C6—H62	107.9	C15—C16—H16	120.0
C6—C7—C8	111.68 (19)	N1—C17—O2	114.38 (19)
C6—C7—H71	109.3	N1—C17—C16	121.8 (2)
C8—C7—H71	109.3	O2—C17—C16	60.66 (16)
C6—C7—H72	109.3	N1—C17—C13	121.21 (19)
C8—C7—H72	109.3	O2—C17—C13	115.49 (19)
H71—C7—H72	107.9	C16—C17—C13	108.68 (19)
C14—C8—C7	111.01 (18)	C13—C18—H181	109.5
C14—C8—C9	107.25 (16)	C13—C18—H182	109.5
C7—C8—C9	110.48 (18)	H181—C18—H182	109.5
C14—C8—H8	109.4	C13—C18—H183	109.5
C7—C8—H8	109.4	H181—C18—H183	109.5
C9—C8—H8	109.4	H182—C18—H183	109.5
C8—C9—C11	111.55 (18)	C10—C19—H191	109.5
C8—C9—C10	114.40 (16)	C10—C19—H192	109.5
C11—C9—C10	113.57 (18)	H191—C19—H192	109.5
C8—C9—H9	105.5	C10—C19—H193	109.5
C11—C9—H9	105.5	H191—C19—H193	109.5
C10—C9—H9	105.5	H192—C19—H193	109.5
C5—C10—C1	109.87 (19)	C21—C20—N1	105.5 (2)
C5—C10—C19	108.59 (17)	C21—C20—H20	127.2
C1—C10—C19	110.26 (19)	N1—C20—H20	127.2
C5—C10—C9	107.71 (17)	C20—C21—N2	111.3 (2)
C1—C10—C9	108.62 (17)	C20—C21—H211	124.3
C19—C10—C9	111.75 (19)	N2—C21—H211	124.3
C12—C11—C9	113.99 (19)	N2—C22—N1	112.2 (3)
C12—C11—H111	108.8	N2—C22—H221	123.9
C9—C11—H111	108.8	N1—C22—H221	123.9
C12—C11—H112	108.8	H31—O3—H32	98.3
			
C10—C1—C2—C3	−54.1 (3)	C17—C13—C14—C8	171.47 (17)
C1—C2—C3—O1	−150.6 (3)	C12—C13—C14—C8	−63.2 (2)
C1—C2—C3—C4	32.9 (3)	C18—C13—C14—C8	59.5 (2)
O1—C3—C4—C5	−179.7 (3)	C17—C13—C14—C15	37.2 (2)
C2—C3—C4—C5	−3.2 (4)	C12—C13—C14—C15	162.61 (19)
C3—C4—C5—C6	171.7 (2)	C18—C13—C14—C15	−74.7 (2)
C3—C4—C5—C10	−7.5 (4)	C8—C14—C15—C16	−167.0 (2)
C4—C5—C6—C7	127.9 (2)	C13—C14—C15—C16	−36.4 (2)
C10—C5—C6—C7	−52.8 (3)	C17—O2—C16—C15	98.7 (2)
C5—C6—C7—C8	53.1 (3)	C14—C15—C16—C17	20.9 (3)
C6—C7—C8—C14	−173.36 (19)	C14—C15—C16—O2	−42.5 (3)
C6—C7—C8—C9	−54.5 (2)	C22—N1—C17—O2	−78.4 (3)
C14—C8—C9—C11	−53.3 (2)	C20—N1—C17—O2	92.2 (3)
C7—C8—C9—C11	−174.37 (19)	C22—N1—C17—C16	−9.0 (4)
C14—C8—C9—C10	176.12 (18)	C20—N1—C17—C16	161.6 (2)
C7—C8—C9—C10	55.0 (3)	C22—N1—C17—C13	135.8 (3)
C4—C5—C10—C1	−12.8 (3)	C20—N1—C17—C13	−53.6 (3)
C6—C5—C10—C1	167.93 (19)	C16—O2—C17—N1	114.2 (2)
C4—C5—C10—C19	107.9 (3)	C16—O2—C17—C13	−98.0 (2)
C6—C5—C10—C19	−71.4 (2)	O2—C16—C17—N1	−102.0 (2)
C4—C5—C10—C9	−130.9 (2)	C15—C16—C17—N1	151.1 (2)
C6—C5—C10—C9	49.8 (2)	C15—C16—C17—O2	−106.8 (2)
C2—C1—C10—C5	43.1 (3)	O2—C16—C17—C13	109.3 (2)
C2—C1—C10—C19	−76.6 (3)	C15—C16—C17—C13	2.5 (3)
C2—C1—C10—C9	160.7 (2)	C12—C13—C17—N1	70.9 (3)
C8—C9—C10—C5	−50.8 (2)	C18—C13—C17—N1	−54.8 (3)
C11—C9—C10—C5	179.61 (18)	C14—C13—C17—N1	−173.19 (19)
C8—C9—C10—C1	−169.70 (19)	C12—C13—C17—O2	−74.6 (3)
C11—C9—C10—C1	60.7 (2)	C18—C13—C17—O2	159.76 (19)
C8—C9—C10—C19	68.4 (2)	C14—C13—C17—O2	41.4 (2)
C11—C9—C10—C19	−61.2 (2)	C12—C13—C17—C16	−140.3 (2)
C8—C9—C11—C12	52.8 (3)	C18—C13—C17—C16	94.1 (2)
C10—C9—C11—C12	−176.12 (19)	C14—C13—C17—C16	−24.3 (2)
C9—C11—C12—C13	−54.5 (3)	C22—N1—C20—C21	−0.2 (3)
C11—C12—C13—C17	168.7 (2)	C17—N1—C20—C21	−172.3 (2)
C11—C12—C13—C18	−68.3 (3)	N1—C20—C21—N2	0.6 (3)
C11—C12—C13—C14	56.2 (2)	C22—N2—C21—C20	−0.7 (3)
C7—C8—C14—C15	−51.4 (3)	C21—N2—C22—N1	0.6 (3)
C9—C8—C14—C15	−172.2 (2)	C20—N1—C22—N2	−0.3 (3)
C7—C8—C14—C13	−178.06 (18)	C17—N1—C22—N2	171.9 (2)
C9—C8—C14—C13	61.2 (2)		

### Hydrogen-bond geometry (Å, º)

**Table:**

*D*—H···*A*	*D*—H	H···*A*	*D*···*A*	*D*—H···*A*
O3—H31···N2	0.90	1.99	2.890 (3)	174
O3—H32···O1^i^	0.90	2.33	3.202 (3)	163
C20—H20···O3^ii^	0.93	2.43	3.208 (4)	142

Symmetry codes: (i) −*x*+3/2, −*y*+1, *z*−1/2; (ii) −*x*+1, *y*−1/2, −*z*+1/2.
